# Experiences and understanding of diagnosis and treatment among drug-resistant extrapulmonary tuberculosis patients: A qualitative study from Central India

**DOI:** 10.1371/journal.pgph.0006383

**Published:** 2026-06-01

**Authors:** Gautam Bhagwat, Pavan Asalampuram, Arti Julka, Cecilia Stålsby Lundborg, Manju Purohit

**Affiliations:** 1 Department of Pathology, R D Gardi Medical College, Ujjain,‌‌ Madhya Pradesh, India; 2 Department of Global Public Health, Karolinska Institutet, Stockholm,‌‌ Sweden; 3 Department of Respiratory Medicine, R D Gardi Medical College, Ujjain, ‌‌Madhya Pradesh, India; Centro de Investigación y Asistencia en Tecnología y Diseño del Estado de Jalisco: Centro de Investigacion y Asistencia en Tecnologia y Diseno del Estado de Jalisco, MEXICO

## Abstract

Extrapulmonary tuberculosis (EPTB) accounts for 15–20% of tuberculosis (TB) cases in immunocompetent individuals and over 50% in people living with Human immunodeficiency virus (HIV). Drug-resistant EPTB presents unique diagnostic and treatment challenges, yet patient experiences remain poorly understood. This study explores how patients with drug-resistant-EPTB perceive and navigate disease acquisition, diagnosis, and treatment within the Indian context. A qualitative study was conducted between January 2022 and July 2024 using in-depth individual interviews with 18 patients diagnosed with drug-resistant-EPTB at a tertiary care centre in Central India. Participants were purposively selected to ensure variation in age, gender, and disease site. Interviews were audio-recorded, transcribed verbatim, and analysed thematically following a structured coding framework. Reporting adhered to COREQ guidelines. Patient experiences were characterized by a “pathway of uncertainty” spanning disease understanding, diagnosis, and treatment. Three major themes emerged. First, limited understanding of EPTB and drug resistance, with misconceptions regarding disease presentation and confusion about the origins of drug resistance, including limited awareness of primary transmission. Second, fragmented diagnostic pathways, marked by delayed diagnosis, multiple healthcare encounters, and reliance on informal care, often compounded by non-specific clinical presentations. Third, uncertainty during treatment, driven by adverse drug effects, lack of clear monitoring indicators, and inadequate communication. These challenges were accompanied by significant socioeconomic and psychosocial impacts, including financial strain, stigma, and gendered vulnerabilities, with some effects persisting beyond treatment. Drug-resistant -EPTB patient experiences reflect interconnected challenges that extend beyond clinical management, forming a continuum of uncertainty across the care pathway. Addressing these requires improved patient-centered communication, strengthened diagnostic pathways, and greater attention to the specific needs of drug-resistant -EPTB within TB control programmes.

## Introduction

Tuberculosis (TB) remains a major global public health challenge. Extrapulmonary tuberculosis (EPTB) accounts for a significant proportion of disease, comprising 15–20% of TB cases in immunocompetent individuals and over 50% among people living with Human immunodeficiency virus (HIV) [1,2]. The emergence and increasing prevalence of multidrug-resistant TB (MDR-TB) and extensively drug-resistant TB (XDR-TB) is a concern to the management of EPTB, especially in resource-limited rural and semi-rural regions [3]. According to the World Health Organization (WHO) Global TB Report 2023, India bears the highest burden of MDR-TB globally, with 22% of all MDR-TB cases worldwide [4]. Drug-resistant EPTB is an undetected, underreported, or misdiagnosed aspect of the TB epidemic. EPTB often involves anatomical sites that are often difficult to access for sample collection, making diagnosis and monitoring more challenging than in pulmonary TB [5]. It further poses unique microbiological confirmation challenge due to the paucibacillary nature of specimens with limited sensitivity of standard diagnostic tools [6]. In addition, treatment regimens are lengthy, toxic, and frequently initiated empirically in the absence of robust microbiological confirmation or drug susceptibility testing [7]. These clinical and programmatic challenges create conditions in which patients may experience fragmented care pathways, delayed diagnosis, and uncertainty regarding their illness and its management.

Despite these complexities, existing research on TB and drug-resistant TB has largely focused on epidemiological patterns, diagnostic performance, and treatment outcomes, with limited attention to how patients interpret, navigate, and respond to these challenges particularly in the context of EPTB. This gap is important because patient understanding, health-seeking behaviour, and interactions with the health system can directly influence diagnostic delays, treatment adherence, and overall outcomes. In EPTB, where symptoms are by definition atypical (without pulmonary presentation) and awareness is low among both among community and healthcare providers, these factors may be especially critical [8]. This results in a system where patients are frequently left confused, unsupported, and at risk of incomplete care. Moreover, knowledge deficits regarding EPTB and its drug-resistant forms among patients and caregivers is compounded by misinformation, cultural misconceptions, and a general lack of health literacy [8–10]. These barriers may negatively impact treatment adherence, psychosocial wellbeing, and overall outcomes [11,12].

In the Indian context, where TB elimination efforts have emphasized expansion of diagnostic services and standardized treatment protocols, there remains limited insight into how patients with drug-resistant-EPTB experience the diagnostic and treatment journey, and how their understanding and beliefs shape engagement with care. Without such insights, programmatic responses risk overlooking key behavioural and system-level barriers that influence outcomes. The aim of this study was to explore the experiences of patients with drug-resistant-EPTB across the continuum of disease acquisition, diagnosis, and treatment, and to identify key factors influencing these experiences, including knowledge, healthcare pathways, and socioeconomic and psychosocial contexts.

## Methodology

### Ethics statement

The study was approved by the Institutional Ethics Committee of R D Gardi Medical College ((Ref: IEC 10/2018). Privacy and confidentiality were strictly maintained throughout the study. Identifying details were removed during transcription, and patients were assigned unique codes for data storage and analysis. Only the research team had access to the recordings and transcripts, which were stored securely on encrypted drives.

### Study design and setting

This hospital-based study employed qualitative research to explore and understand the lived experiences, perceptions, and treatment practices of patients diagnosed with drug-resistant-EPTB. The study followed the consolidated criteria for reporting qualitative research (COREQ) guidelines (S1 Checklist) to ensure methodological rigor and transparency [13].

The study was conducted at Central Research Laboratory, Ruxmaniben Deep Chand Gardi Medical College (RDGMC), a tertiary care teaching institute with associated Chandrikaben Rashmikant Gardi Hospital (CRGH), and associated rural outreach center in Ujjain, Central India between January 2022 to July 2024. The hospital serves surrounding high TB burden rural and semi-urban villages, and caters to patients from low- to middle-income backgrounds. CRGH is a nodal center for diagnosis and management for both pulmonary and extrapulmonary forms of TB and drug-resistant-TB patients of the Ujjain Commissionaire (seven districts-90,74,036 population) under the National Tuberculosis Elimination Program (NTEP) that provides second-line treatment, Bedaquiline, Pretomanid, Linezolid, and Moxifloxacin (BPLaM) therapy and counselling services for patients diagnosed with drug-resistant-TB. The hospital has a modest outpatient department, inpatient wards, dedicated drug resistant-TB ward, advanced diagnostic laboratory facilities including GeneXpert, biopsy-based investigations and imaging facilities.

### Patients

Patients were enrolled for individual interview from out- and in-patient services, using a strategic sampling approach due to the limited and specialized nature of the drug-resistant-EPTB patient pool. The target population comprised patients diagnosed with drug-resistant-EPTB. Eligibility criteria included individuals aged >14 years, confirmed to have drug-resistant-EPTB and who were either undergoing treatment at the time of the study or had completed treatment within the previous 12 months under NTEP guidelines. Drug-resistant -EPTB was defined as EPTB confirmed via microbiological testing (e.g., CBNAAT/Xpert MTB/RIF, Line Probe Assay, or culture-based drug susceptibility testing) with documented resistance to at least rifampicin or additional first/second-line anti-TB drugs. Patients with cognitive impairments, or severe illness at the time of recruitment were excluded. Eligible patients were identified with the assistance of the hospital’s DOTS coordinator, chest physician, and medical records department. Potential patients were approached either during their scheduled outpatient follow-up visits or during hospitalization. No financial incentives were provided. A total of 18 patients participated in the study.

### Data collection

An open-ended, semi-structured interview guide was developed collaboratively by a multidisciplinary team comprising, infectious disease specialists, and social scientists (S1 Text). The guide was piloted among three drug-resistant-EPTB patients (not included in the data analysis) and refined accordingly. The finalized guide consisted of two major aspects 1. Cause and Transmission of drug-resistant-TB: Questions focused on the patient’s knowledge of disease acquisition, potential sources of infection (household, community, healthcare), and history of exposure to known TB cases. Probes explored the nature and duration of exposure, TB type in index cases (drug-sensitive or resistant), and the diagnostic and treatment history of the presumed source. 2. Diagnosis and Treatment as a Cause of drug-resistant-TB: Questions assessed treatment-seeking behavior, diagnostic delays, adherence, side effects, drug availability, treatment interruptions, and use of formal v/s informal care providers. The additional domains were also included as-1. Personal factors (knowledge, beliefs, attitudes, conceptualization of illness), 2. External factors (healthcare organization, financial barriers, structural factors), 3. Support systems (family, social stigma, media, community influence), 4. Unanticipated issues (e.g., comorbidities, misinformation, mobility patterns).

Interviews were conducted by trained health professional in a private room within the hospital premises. The study purpose, procedures, and confidentiality aspects were explained in detail by the investigator in the local language (Hindi). Prior to each interview, written informed consent was obtained from all patients. Patients were informed that participation was voluntary and that declining or withdrawing would not affect their care. Each interview lasted approximately 40–60 minutes and was audio-recorded with the patient’s consent. The interviews were conducted in Hindi or a local dialect, and field notes were taken to capture non-verbal cues and contextual information. The interviewers adhered to a non-judgmental and empathetic approach, allowing the patients to speak freely. Interviews continued until thematic saturation was reached, defined as the point at which no new themes emerged from subsequent interviews. A total of 18 interviews were completed, with saturation achieved after 16 patients.

### Analysis

All interviews were transcribed verbatim in Hindi and translated into English by the authors. The analysis was performed manually. To maintain accuracy and fidelity to the original meaning, 20% of the English translations were back-translated by an independent bilingual translator. The research team reviewed each transcript alongside the audio recordings to ensure completeness and to make corrections for clarity and language meaning, especially where idiomatic expressions were used. Each patient was assigned a unique identifier to ensure anonymity during analysis and reporting. Data were analyzed using thematic content analysis. Transcripts were coded inductively, and emerging themes were discussed among the research team to ensure consistency and reliability. The analysis followed the six-step framework outlined by Braun and Clarke [14]. The process began with data familiarization of meaning units through repeated reading of the transcripts to condensed units and initial coding was conducted inductively, with codes representing units of meaning relevant to the research objectives. These codes were then reviewed, refined, and grouped into broader (sub) categories and (sub) themes that captured underlying patterns in patients’ narratives. The coding process was iterative, with regular team discussions to revise the codebook and ensure consistency. Two members of the research team independently coded a subset of transcripts, and discrepancies were resolved through consensus. The final (sub) themes were selected based on relevance, recurrence, and explanatory power.

To enhance credibility, the study followed strategies such as prolonged engagement with patients, peer debriefing among research team members, and triangulation of data sources including interview transcripts and field notes. Transferability was supported by providing detailed descriptions of the study setting, patient characteristics, and context of TB care in Central India. Dependability and confirmability were ensured by maintaining an audit trail that documented decisions regarding sampling, coding, and theme development. Reflexivity was practiced throughout the study by maintaining a notebook to note assumptions and probing during the interview interaction with patient. While every effort was made to reduce subjectivity, the interpretive nature of qualitative analysis was acknowledged, and transparency was prioritized in reporting. This study was linked to a larger quantitative cohort and the insights were interpreted with reference to NTEP guidelines, community health systems, and socio-economic realities in Central India.

## Results

### Overview of patients

A total of 21 patients with drug-resistant-EPTB were identified and approached for the study. One patient was not included due to not meeting the age-related inclusion criteria (10 years old), and two patients declined to participate. Individual-interviews were conducted with 18 patients. The patients represented a diverse range of ages (15–52 years), gender (12 females, 6 males), and EPTB sites (lymph node, pleura, spine, abdominal, and breast TB).

Most participants were newly diagnosed cases, with only a small proportion reporting a prior history of TB treatment. Participants represented diverse socioeconomic backgrounds, with many engaged in informal or daily-wage occupations, and a subset reporting limited or no regular income during the illness period. Educational status varied, with several participants having limited formal education, which appeared to influence their understanding of disease and treatment. A detailed summary of participant characteristics is presented in Table 1 and S1 Table.

**Table 1:** Clinical Characteristics of Interviewed drug-resistant-EPTB Patients.

Patient ID	Age (years)	Gender	New/previously treated	Sample type	Site of EPTB	Education level	Occupation
P1	52	M	N	Pus	Spine	Primary	Tea Stall Vendor
P2	50	M	N	Fluid	Pleural	Primary	Rickshaw driver
P3	20	F	N	FNAC	Lymph node	Secondary	Homemaker
P4	20	F	N	Fluid	Pleural	Higher secondary	Student
P5	30	F	N	Fluid	Abdominal	Higher secondary	Student
P6	25	F	N	Pus	Cervical Lymph node	Higher secondary	Homemaker
P7	26	F	N	Lymph node-tissue	Cervical Lymph node	Graduate	School Teacher
P8	27	F	N	Lymph node	Cervical Lymph node	Primary	Domestic Helper
P9	25	F	N	Fluid	Pleural	Primary	Homemaker
P10	34	F	N	Pus	Abdominal	Higher secondary	Farm Labourer
P11	36	M	N	FNAC aspirate	Cervical lymph node	Secondar	Security Guard
P12	45	M	N	fluid	Pleural	Secondary	Farmer
P13	50	F	N	Pus	Cervical lymph node	Secondary	Home maker
P14	38	M	PT	Pus	Spine	Higher secondary	Shopkeeper
P15	18	F	N	fluid	Pleural	Secondary	None
P16	18	M	N	fluid	Pleural	Higher secondary	Student
P17	18	F	PT	Tissue	Breast	Secondary	Student
P18	27	F	N	Pus	Lymph node	Graduate	Homemaker

**Fig 1:**
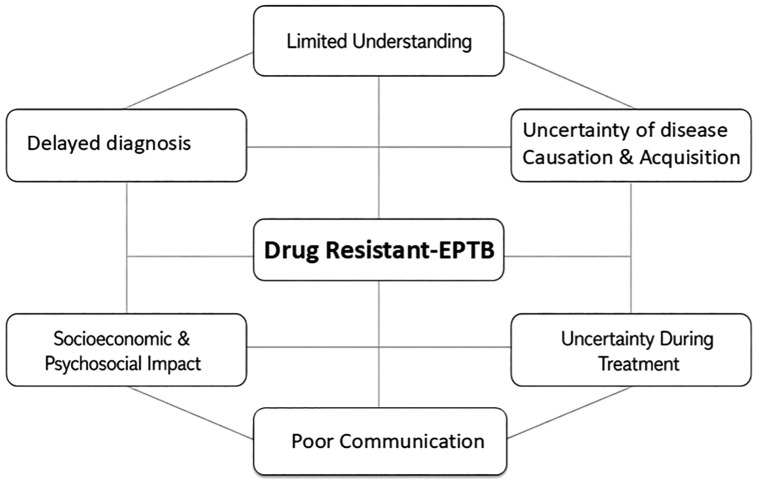
Key domains of patients experience in drug-resistant -EPTB identified through thematic analysis of in-depth interviews. The domains are inter-related and may overlap rather than fixed and linear consequence.

Analysis of participant narratives suggests that experiences of drug-resistant-EPTB can be understood as a “pathway of uncertainty”, spanning from limited understanding of disease to challenges in diagnosis and treatment. These stages were interconnected and shaped by broader social and structural factors represented as themes (Fig 1). Each theme includes key subthemes supported by illustrative patient quotes as follows (S2 Table).

### Theme 1: Limited understanding of EPTB and drug resistance

Patients’ understanding of how they acquired drug resistant-EPTB was shaped by limited biomedical knowledge, social interpretations of illness, and prior experiences with TB. Most participants were unable to clearly identify the source of infection, and their explanations often reflected uncertainty and misconceptions.

#### Subtheme 1.1: Misconceptions about EPTB and disease causation.

Many participants were unaware that TB could affect organs other than the lungs. TB was commonly perceived as a respiratory disease associated with cough, weight loss, and infectiousness. As a result, extrapulmonary manifestations were often not recognized as TB, leading to confusion during early illness.

*“I didn’t think it was TB because I was not coughing like others… I had no idea TB can be like this—without cough, without fever even.”* (Female, lymph node TB, 26 years)

In addition to limited awareness of EPTB, several participants attributed their illness to non-biomedical causes such as food habits, hygiene, or personal weakness. These beliefs reflected broader social and cultural interpretations of illness.

*“People around me kept saying I got this illness because I often eat outside… some even said it’s because I don’t follow proper cleanliness.”* (Female, abdominal TB, 20 years)

Understanding of drug resistance was also limited. Most participants were unfamiliar with the distinction between drug-sensitive and drug-resistant TB prior to diagnosis. Among those with previous TB treatment, incomplete adherence was sometimes retrospectively linked to their current illness. In contrast, participants without prior TB history expressed confusion about how they developed drug-resistant disease.

*“This is my first time with TB. I never had any symptoms before, never took any TB medicines in my life. But now they are saying I have* drug-resistant *-TB. I don’t understand—I’m scared because everyone says drug-resistant -TB is more dangerous and difficult to cure, though three members of our family members got TB but I am not sure if they also had* drug-resistant *type.”* (Male, lymph node TB, 36 years)

These findings suggest that misconceptions extended beyond disease recognition to include the origins and implications of drug resistance.

#### Subtheme 1.2: Uncertain and varied interpretations of infection source.

Despite limited understanding, some participants attempted to identify possible sources of infection. These interpretations were often based on household exposure, community contact, or interactions within healthcare settings.

Several participants reported prior contact with family members who had TB and considered this a potential source of infection, although the mechanisms of transmission were not clearly understood.

*“My husband had TB around two years ago… I keep wondering—did I get it from him?”* (Female, abdominal TB, 42 years)

Others attributed their illness to community-level exposure, including contact with individuals in villages or healthcare facilities. Overcrowded hospital environments, particularly waiting areas with coughing patients, were frequently perceived as potential sites of infection.

*“We sat for hours in the hospital… there were so many patients coughing around… I keep thinking—did I catch it that day?”* (Male, pleural TB, 50 years)

However, even when participants identified possible exposures, understanding of how extrapulmonary disease develops or how drug resistance occurs remained unclear. These narratives highlight a combination of partial awareness and persistent uncertainty regarding disease acquisition.

### Theme 2: diagnostic pathways and uncertainty during treatment

Participants described a complex and often prolonged journey through the healthcare system, characterized by delayed diagnosis, multiple provider interactions, and challenges during treatment. These experiences contributed to uncertainty across the care pathway and influenced patient engagement with treatment.

#### Subtheme 2.1: diagnostic pathways.

Many participants reported prolonged symptom duration before receiving a definitive diagnosis. Initial consultations were often with informal providers, private practitioners, or local chemists, where treatment focused on symptom relief rather than diagnostic evaluation for TB.

*“For months, they gave me medicines for fever and pain. No one tested me for TB. Only when I got very weak, I was referred to the big hospital.”* (Male, bone TB, 38 years)

Patients frequently described multiple healthcare visits and misdiagnoses, particularly due to the non-specific presentation of EPTB. In several cases, diagnostic delays were further compounded by limited access to site-specific investigations such as biopsy or imaging.

*“They said it’s a gland disease and gave me injections… after four months it became worse. Only then someone sent me for a TB test.”* (Female, lymph node TB, 20 years)

*“I never imagined TB could be in the spine without cough or fever… it took months before they did an MRI.”* (Male, spine TB, 38 years)

Participants also reported challenges navigating the healthcare system, including repeated referrals, delays in receiving laboratory results, and logistical barriers related to travel and access.

*“I had to go three times just to get the report… coming from the village wasn’t easy… by then, my condition had worsened.”* (Female, abdominal TB, 34 years)

In some cases, participants described the use of antibiotics or other medications prior to diagnosis, often obtained from informal providers or over-the-counter sources, which may have masked symptoms and delayed appropriate testing.

*“Before I was diagnosed, I used to take antibiotics from the medical store… my pain reduced for some time.”* (Female, lymph node TB, 50 years)

These findings indicate that diagnostic delays in drug-resistant-EPTB were shaped by both health system factors and patterns of healthcare-seeking behaviour.

#### Subtheme 2.2: Treatment challenges, communication and monitoring.

Participants reported multiple challenges during the treatment phase, including difficulties with adherence, adverse drug effects, and uncertainty regarding treatment progress. Side effects such as vomiting, weakness, and gastrointestinal discomfort were frequently described and, in some cases, led to treatment interruption.

*“The medicine made me vomit… I kept feeling weak… I just decided to stop on my own.”* (Female, breast TB, 18 years)

A prominent concern was the lack of clear understanding of treatment response. Unlike pulmonary TB, participants described the absence of visible or measurable indicators of improvement, which contributed to confusion and reduced confidence in treatment.

*“There was no cough or sputum… I kept wondering how do they know I’m getting better?”* (Male, spine TB, 38 years)

*“I kept taking the drugs, but I didn’t know if it was working… there was nothing to show if I was cured or not.”* (Female, lymph node TB, 26 years)

In addition, participants highlighted gaps in communication within the healthcare system. Several reported not being adequately informed about the nature of drug-resistant TB, changes in treatment regimens, or the expected duration and outcomes of therapy.

*“No one told me what drug-resistant means… they just gave a different set of medicines.”* (Female, lymph node TB, 27 years)

Discontinuity of care between private and public sectors further contributed to confusion, particularly when treatment regimens were modified without clear explanation.

*“They just changed the medicine… I didn’t understand why this time it was different.”* (Female, abdominal TB, 34 years)

Overall, these experiences reflect a broader pattern of uncertainty during treatment, shaped by side effects, inadequate communication, and lack of clear monitoring indicators.

### Theme 3: Socioeconomic and psychosocial impact across the illness trajectory

Participants described substantial socioeconomic and psychosocial impacts associated with drug-resistant-EPTB, most prominently during the active phase of illness and treatment. However, some narratives suggested that financial strain and social consequences could persist beyond treatment, indicating potential longer-term effects.

#### Subtheme 3.1: Financial strain and work disruption during illness, with lingering consequences.

The illness and prolonged treatment period resulted in a significant loss of livelihood, particularly among daily-wage workers and individuals engaged in informal employment. Many participants reported being unable to work for extended periods, leading to financial instability, depletion of savings, and debt.

*“I couldn’t go to work for two months; all my money was gone… I had to borrow from neighbours, and I still don’t know how I’ll repay them. This illness doesn’t just break the body—it breaks the whole family.”* (Male, spine TB, 38 years)

Some participants described selling household assets or becoming dependent on relatives for basic needs such as food, medicines, and daily activities. While these impacts were most pronounced during the treatment phase, a few participants indicated that financial difficulties persisted beyond treatment, particularly in the form of ongoing debt and reduced earning capacity.

#### Subtheme 3.2: Emotional distress, stigma, and gendered experiences.

Participants frequently reported emotional distress, stigma, and social isolation, particularly during the period of active illness. Many described being avoided by neighbours, extended family members, and, in some cases, colleagues, especially after disclosure of their diagnosis.

*“Even in this age of mobile phones, people in the village still react with fear when they hear the name ‘TB’… people’s behaviour changes… TB patients are seen as somehow lesser.”* (Female, lymph node TB, 26 years)

Participants expressed feelings of shame, fear, and emotional exhaustion, often linked to perceived social judgement and changes in interpersonal relationships.

*“My brother used to talk to me daily but now started keeping their distance… I began to feel like I was different—as if I wasn’t just sick but tainted.”* (Female, lymph node TB, 27 years)

In some cases, these experiences appeared to persist beyond the treatment period, particularly where stigma or altered social relationships remained unresolved.

Gender emerged as a cross-cutting factor influencing these experiences. Female participants described delays in seeking care due to household responsibilities and fear of social consequences, particularly within marital families. Concerns about disclosure and social acceptance were prominent.

*“I was scared to tell anyone… I worried my in-laws might not take it well… for a woman, it’s very difficult to talk about these things.”* (Female, abdominal TB, 30 years)

Male participants, in contrast, more frequently emphasized the psychological impact of financial loss and inability to fulfil expected roles as primary earners.

## Discussion

This study provides in-depth insight into how patients with drug-resistant EPTB experience and interpret the processes of disease acquisition, diagnosis, and treatment. Rather than representing isolated barriers, the findings suggest a connected “pathway of uncertainty,” in which limited understanding of disease, fragmented diagnostic pathways, and ambiguity during treatment interact to shape patient trajectories and engagement with care. Through iterative thematic analysis, these domains were found to reinforce one another, producing a compounded experience that is distinct from what is typically described in either EPTB or pulmonary drug-resistant TB alone. Taken together, these findings suggest that uncertainty functions not as an isolated experience but as a cumulative process spanning disease recognition, diagnosis, treatment interpretation, and future expectations.

### Understanding of EPTB and drug resistance: misconceptions and implications

A key finding of this study is the limited and often inaccurate understanding of EPTB and drug resistance among patients. Rather than isolated misconceptions, the findings point to a broader structural gap in how EPTB is conceptualised at the community level, where TB continues to be understood primarily as a pulmonary disease. Similar gaps in awareness have been reported in previous studies [8–10,15], but our findings extend this evidence by demonstrating that misrecognition persists even after diagnosis, influencing how patients interpret disease severity and the necessity of prolonged treatment.

Importantly, misconceptions extended to the understanding of drug resistance. The dominance of an “acquired resistance” narrative among participants despite most being newly diagnosed highlights a misalignment between patient understanding and evolving epidemiology. In the present study, the predominance of newly diagnosed cases (16 out of 18) raises the possibility that transmission of resistant strains may be more important than commonly perceived. While this observation is based on a small qualitative sample and should be interpreted cautiously, it aligns with emerging epidemiological evidence from India and other high-burden settings demonstrating a substantial proportion of primary drug-resistant TB, including among extrapulmonary cases [5, 16,17]. Previous molecular epidemiology and programmatic data have shown that a considerable proportion of drug-resistant TB cases occur in patients without prior treatment exposure, supporting ongoing transmission of resistant strains rather than resistance arising solely due to inadequate therapy. These findings suggest that primary transmission is not only under-recognised epidemiologically but also conceptually invisible to patients, which may influence perceived susceptibility and treatment adherence.

### Diagnostic pathways: beyond availability of diagnostics

Consistent with studies from India and sub-Saharan Africa, prolonged and complex diagnostic journeys characterised by multiple consultations, misdiagnoses, and delayed referrals appear to persist despite expanded molecular testing. There is still a gap between the availability of diagnostic technologies and their effective utilisation [5, 9, 11, 16–18]. Despite substantial expansion of rapid molecular diagnostics such as CBNAAT (GeneXpert) and Truenat across India, diagnostic delays persist [19]. In the context of EPTB, these challenges are amplified by non-specific clinical presentations, difficulties in obtaining appropriate specimens, and the need for site-specific investigations [9,20].

Compared to pulmonary TB, where symptom recognition and sputum-based testing provide clearer entry points into care, EPTB lacks standardised and easily accessible diagnostic pathways [8,9,20]. The addition of drug resistance further complicates this trajectory, requiring layered testing that is often delayed or fragmented. Pre-diagnostic antibiotic use observed in this study reflects not only patient behaviour but also systemic diagnostic uncertainty, a feature widely described in TB care pathways [20,21]. While pulmonary drug-resistant-TB programmes increasingly rely on algorithm-driven testing, drug-resistant-EPTB appears to remain diagnostically less structured. Both patient-related and health system delays continue to contribute to prolonged time to diagnosis among EPTB patients, underscoring the need for more integrated and streamlined diagnostic approaches [9,17,18].

### Uncertainty during treatment: monitoring, communication, and adherence

Uncertainty regarding disease progression and treatment response emerged as a central feature of patient experience, particularly regarding disease progression and response to therapy [16]. Unlike pulmonary TB, where sputum conversion provides a tangible and widely understood indicator of improvement, patients with EPTB largely rely on subjective symptoms that may fluctuate or resolve slowly, making treatment response difficult to interpret [5,22].

This lack of clear, patient-understandable markers contributed to confusion and reduced confidence in treatment, and in some cases led to treatment interruption. These challenges were further compounded by adverse drug effects, including vomiting and fatigue, particularly when not adequately anticipated or managed [5,11,23–25]. While previous studies have highlighted the influence of treatment duration, pill burden, and side effects on adherence [15,24], in drug-resistant-EPTB, adherence is shaped not only by regimen complexity but also by the interpretability of treatment response [5,22,26].

Gaps in communication regarding diagnosis, treatment changes, and the implications of drug resistance further intensified this uncertainty. Inconsistent messaging, particularly across private and public healthcare sectors, contributed to patient confusion and fragmented understanding of care. Taken together, these findings indicate that, unlike pulmonary drug-resistant TB where adherence barriers are often predominantly regimen-driven, in drug-resistant-EPTB they are strongly mediated by uncertainty arising from both limited ‌‌monitoring clarity and inadequate communication.

### Socioeconomic and psychosocial impact: acute burden with potential long-term effects

The socioeconomic and psychosocial impacts of drug-resistant-EPTB were substantial and most pronounced during the active phase of illness and treatment. These findings are consistent with previous literature demonstrating the economic burden of TB, even where treatment is provided free of cost [27–29]. However, the persistence of financial strain beyond treatment in some participants suggests that the impact of drug-resistant-EPTB may extend into post-treatment recovery, a dimension described in few EPTB-focused studies [27]. Similar psychosocial experiences, including anticipated stigma, social isolation, and delayed care-seeking, have also been described in qualitative studies from South Africa and Ethiopia, suggesting that TB-related stigma persists across diverse epidemiological and cultural settings despite differences in disease presentation and infectiousness.

The diagnosis of drug resistance further contributed to psychological distress, driven by stigma, fear of infecting family members, uncertainty about the future, and reduced ability to perform daily activities [30]. Stigma and social isolation were prominent despite EPTB being less infectious than pulmonary TB, indicating that TB-related stigma operates independently of actual transmission risk and is instead shaped by entrenched social perceptions of the disease. While stigma is well documented in pulmonary TB [31], its persistence in EPTB highlights the generalisation of TB-related social narratives beyond clinical infectiousness.

Gender differences further influenced these experiences. While consistent with existing literature [32], the findings suggest that gendered impacts intersect with disease form—where the relative invisibility of EPTB symptoms may delay recognition among women, while prolonged morbidity may more directly affect economic roles among men.

### Implications for drug-resistant-EPTB care within TB control programmes

A key finding of this study is that the experience of drug-resistant-EPTB differs from pulmonary drug-resistant TB in several important ways. Unlike pulmonary drug resistant-TB, where sputum conversion and cough reduction provide visible indicators of treatment response, patients with drug-resistant-EPTB often lack clear or patient-understandable markers of improvement, contributing to uncertainty regarding treatment effectiveness [9,22]. The non-specific and frequently non-visible nature of EPTB symptoms also contributes to delayed recognition and fragmented diagnostic pathways requiring multiple referrals and invasive investigations [8,9,18]. Furthermore, despite generally lower infectiousness, patients continued to experience substantial stigma, suggesting that TB-related social perceptions extend beyond actual transmission risk [30,31]. Together, these findings indicate that drug-resistant-EPTB is characterised not only by treatment complexity but also by diagnostic ambiguity and difficulties in interpreting treatment response and recovery, distinguishing it from the more structured clinical experience of pulmonary drug-resistant TB.

These findings highlight the need for more integrated and patient-centred approaches for drug-resistant-EPTB within TB control programmes. Strengthening EPTB-specific counselling at diagnosis, streamlining referral pathways, and improving coordination between private and public healthcare sectors may help reduce diagnostic delays and patient uncertainty. In addition, structured symptom-based follow-up and clearer communication regarding treatment progress may improve patient understanding, confidence, and adherence. These findings further support WHO recommendations advocating people-centred TB care models that incorporate communication, psychosocial support, and patient-understandable treatment pathways alongside biomedical management [33].

### Strengths and limitations

A key strength of this study is the in-depth exploration of patient experiences in a relatively under-researched population of drug-resistant-EPTB patients. The use of individual interviews enabled detailed insights across the diagnostic and treatment continuum. Data were collected prospectively using a structured interview guide, and measures such as peer debriefing and transcript review were employed to enhance analytical rigour.

However, several limitations should be considered. As a single-centre qualitative study, the findings may not be fully transferable to other settings. Participants were recruited after diagnosis, and their accounts may be subject to recall bias. Additionally, the study focuses exclusively on patient perspectives and does not include healthcare provider or undiagnosed patient viewpoints, which may further contextualise the findings.

## Conclusion

Patients with drug-resistant-EPTB experience a complex and interconnected set of challenges spanning disease understanding, diagnosis, and treatment. These challenges form a continuum of uncertainty that shapes patient engagement and influences treatment outcomes. Addressing these gaps requires not only improvements in diagnostic and treatment delivery systems but also greater emphasis on patient understanding, communication, and continuity of care. Integrating these patient-centred considerations into TB control strategies will be essential for‌‌ effectively addressing drug-resistant-EPTB within ongoing TB elimination efforts.

## Supporting information

S1 Checklist Consolidated criteria for reporting qualitative research (COREQ) checklist.
(DOCX)

S1 Text Interview guide with probes.
(DOCX)

S1 Table Sociodemographic and clinical characteristics of interviewed drug-resistant-EPTB Patients.
(DOCX)

S2 Table Thematic structure-experience from individual interviews with drug-resistant -EPTB patients with data set.
(DOCX)

S1 Data Data.
(XLSX)
