# Supplementary material for: Experiences and understanding of diagnosis and treatment among drug-resistant extrapulmonary tuberculosis patients: A qualitative study from Central India
Source: PLOS Glob Public Health. 2026 Jun 1;6(6):e0006383. doi: 10.1371/journal.pgph.0006383 (PMC13225431; doi:10.1371/journal.pgph.0006383)
Supplement: S1 Checklist — Consolidated criteria for reporting qualitative research (COREQ) checklist. (DOCX) [file pgph.0006383.s001.docx]

S1 Checklist. COREQ Checklist (Consolidated Criteria for Reporting Qualitative Research)

Domain 1: Research team and reflexivity

| **Item** | **Description** | **Included in manuscript** |
| --- | --- | --- |
| 1 | Interviewer/facilitator | Yes – ID specialists and trained qualitative researchers conducted interviews |
| 2 | Credentials | Yes – Authors’ degrees and roles listed |
| 3 | Occupation | Yes – Clinical and academic |
| 4 | Gender | Yes – Implied but can be clarified |
| 5 | Experience and training | Yes – Mentioned qualitative training and pilot testing |
| 6 | Relationship established | Yes – Rapport building discussed in methods |
| 7 | Patient knowledge of interviewer | Yes – Patients informed about research aims |
| 8 | Interviewer characteristics | Partially – Consider expanding on reflexivity |

Domain 2: Study design

| **Item** | **Description** | **Included in manuscript** |
| --- | --- | --- |
| 9 | Methodological orientation | Yes – Thematic analysis |
| 10 | Sampling | Yes – convenience sampling |
| 11 | Method of approach | Yes – In-person approach during treatment |
| 12 | Sample size | Yes – 18 DR-EPTB patients |
| 13 | Non-participation | Yes – 2 declined; reason briefly mentioned |
| 14 | Setting of data collection | Yes – Private hospital or clinic room |
| 15 | Presence of non-patients | Yes – No others present during interviews |
| 16 | Description of sample | Yes – Sociodemographic table provided |
| 17 | Interview guide | Yes – Developed and refined; provided in supplement |
| 18 | Repeat interviews | No – Not conducted |
| 19 | Audio/visual recording | Yes – Audio-recorded with consent |
| 20 | Field notes | Yes – Taken alongside interviews |
| 21 | Duration | Yes – 40–60 minutes |
| 22 | Data saturation | Yes – Mentioned saturation reached by 16 interviews |
| 23 | Transcripts returned | No – Not returned for validation |

Domain 3: Analysis and findings

| **Item** | **Description** | **Included in manuscript** |
| --- | --- | --- |
| 24 | Number of data coders | Yes – 2 coders, reviewed jointly |
| 25 | Description of coding tree | Yes – Codebook/table provided |
| 26 | Derivation of themes | Yes – Inductive thematic analysis |
| 27 | Software | Yes – manual |
| 28 | Patient checking | No – Not done post-interview |
| 29 | Quotations presented | Yes – Rich, long quotes with translation |
| 30 | Data and findings consistency | Yes – Clear mapping between quotes and themes |
| 31 | Clarity of major themes | Yes – Strong structure around themes/subthemes |
| 32 | Clarity of minor themes | Yes – Cross cutting findings (gender) discussed |
