# Supplementary material for: Experiences and understanding of diagnosis and treatment among drug-resistant extrapulmonary tuberculosis patients: A qualitative study from Central India
Source: PLOS Glob Public Health. 2026 Jun 1;6(6):e0006383. doi: 10.1371/journal.pgph.0006383 (PMC13225431; doi:10.1371/journal.pgph.0006383)
Supplement: S1 Text — Interview guide with probes. (DOCX) [file pgph.0006383.s002.docx]

S1 Text: Interview Guide with Probes

**In-Depth Interview Guide**

Study Title: *Perception and Practice of Drug-Resistant Extrapulmonary Tuberculosis Patients: A Qualitative Analysis from Central India*
Target Patients: Diagnosed DR-EPTB patients (≥14 years) who have completed or are undergoing treatment.
Method: Semi-structured in-depth interview
Tool Type: Open-ended, with prompts and probes for deeper exploration

Section A: Introduction & Rapport Building

- Can you tell me a bit about yourself? (age, work, family)
- How did you first start feeling unwell? What symptoms did you have?

Section B: Understanding and Perceptions of EPTB and DR-TB

1. Have you heard of tuberculosis (TB) before you were diagnosed?
   1. What did you think TB was?
   2. Did you know TB can happen outside the lungs?
2. Before your illness, had you heard of drug-resistant TB (DR-TB)?
   1. What do you think causes TB to become drug-resistant?
   2. Where did you hear or learn about TB or DR-TB?

*Probes: Knowledge sources (community, media, health workers), misconceptions, traditional beliefs*

Section C: Diagnostic Journey

1. Can you describe your experience from when you first noticed symptoms to when you were diagnosed?
   1. What symptoms did you have and when did they start?
   2. Where did you go first for help?
   3. How many doctors or hospitals did you visit before diagnosis?
   4. Were any tests done before DR-TB was confirmed?
2. How did you feel during this period of not knowing what was wrong?

*Probes: Delay in diagnosis, misdiagnosis, cost of multiple visits, emotional toll*

Section D: Treatment Experience

1. Once diagnosed, how did the treatment process begin?
   1. Did you face any difficulties in starting treatment?
   2. Were medicines always available?
   3. Did anyone explain how long the treatment would be?
2. How has the treatment affected you physically?
   1. Did you have any side effects?
   2. Were you able to continue the full course?
3. What helped or made it difficult to continue the treatment?

*Probes: Access issues, interruptions, side effects, support, counselling*

Section E: Social and Psychological Impact

1. How has this illness affected your daily life and relationships?
   1. Has your role in the family changed?
   2. How have your friends, neighbours, or coworkers reacted?
   3. Did you feel you were treated differently by others?
2. How did you feel emotionally during treatment?
   1. Did you feel scared, depressed, hopeful?

*Probes: Isolation, stigma, disclosure concerns, community support*

Section F: Economic Burden and Support

1. Has this illness affected your income or financial situation?
   1. Were you able to continue working?
   2. Did any family member stop working to care for you?
2. Did you receive any financial support from government or others?
   1. Was it sufficient?
   2. Did you have to borrow money or sell assets?

*Probes: Nikshay Poshan Yojana, other welfare schemes, out-of-pocket expenses*

Section G: Health System Experience

1. How did you find the overall healthcare services during your illness?
   1. Were doctors/nurses supportive?
   2. Did you understand the instructions you were given?
   3. Were follow-ups and counselling sessions available?
2. What was your experience with getting referred or moved between hospitals (if any)?

*Probes: Navigation difficulties, communication issues, trust in system*

Section H: Suggestions and Reflections

1. What could be done to help other patients with drug-resistant TB like you?
   1. What support do patients need most?
   2. What would you improve in the TB program?
2. Is there anything else you would like to share about your experience that we haven’t covered?

Notes for Interviewer:

- Be flexible. Let the patient speak freely and modify probes as needed.
- Use non-judgmental language; ensure patient comfort and privacy.
- Always ask permission before recording or noting sensitive content.
- Always complete the interview with thanking the patient.
