# Supplementary material for: Experiences and understanding of diagnosis and treatment among drug-resistant extrapulmonary tuberculosis patients: A qualitative study from Central India
Source: PLOS Glob Public Health. 2026 Jun 1;6(6):e0006383. doi: 10.1371/journal.pgph.0006383 (PMC13225431; doi:10.1371/journal.pgph.0006383)
Supplement: S1 Table — Sociodemographic and clinical characteristics of interviewed drug-resistant-EPTB Patients. (DOCX) [file pgph.0006383.s003.docx]

**S1 Table. Sociodemographic and Clinical Characteristics of Interviewed DR-EPTB Patients (n = 18)**

| **Characteristic** | **Category** | **n (%)** |
| --- | --- | --- |
| **Age Group (years)** | 18–29 | 10 (55) |
|  | 30–49 | 05 (28) |
|  | ≥50 | 3 (17) |
| **Gender** | Male | 06 (33) |
|  | Female | 12 (67) |
| **Education Level** | No formal education | 0 |
|  | Primary (1–5 class) | 4 (23) |
|  | Secondary (6–10 class) | 6 (33) |
|  | Higher secondary and above | 8 (44) |
| **Occupation** | Daily wage labourer | 2 (11) |
|  | Farmer | 1 (6) |
|  | Homemaker | 5 (28) |
|  | Other (driver, shopkeeper, etc.) | 6 (33) |
|  | Student | 4 (22) |
| **Monthly Household Income** | < ₹5000 | 2 (11) |
|  | ₹5000–₹10,000 | 10 (55) |
|  | > ₹10,000 | 6 (33) |
| **Marital Status** | Married | 11 (39) |
|  | Unmarried | 7 (33) |
| **Site of EPTB** | Pleural TB | 6 (33) |
|  | Lymph node TB | 7 (39) |
|  | Spinal TB | 2 (11) |
|  | Abdominal TB | 2 (11) |
|  | Breast | 1 (6) |
| **Previous History of TB Treatment** | No | 16 (89) |
|  | Yes | 02 (11) |
| **Comorbidity (e.g., Diabetes)** | Yes | 1 (6) |
|  | No | 17 (94) |
