# Supplementary material for: Experiences and understanding of diagnosis and treatment among drug-resistant extrapulmonary tuberculosis patients: A qualitative study from Central India
Source: PLOS Glob Public Health. 2026 Jun 1;6(6):e0006383. doi: 10.1371/journal.pgph.0006383 (PMC13225431; doi:10.1371/journal.pgph.0006383)
Supplement: S2 Table — Thematic structure-experience from individual interviews with drug-resistant -EPTB patients with data set. (DOCX) [file pgph.0006383.s004.docx]

S2 Table: Thematic Structure of DR-EPTB Illness Experience from Individual Interviews with DR-EPTB Patients

| **Theme** | **Subtheme** | **Category** | **Meaning Unit (Verbatim Quote)** |
| --- | --- | --- | --- |
| **Theme 1: Limited understanding of EPTB and drug resistance** | **1.1 Misconception about EPTB and disease causation** | Knowledge gaps | “I didn’t think it was TB because I was not coughing like others… I had no idea TB can be like this—without cough, without fever even.”  “I didn’t know TB could happen in the back. I thought TB only means cough.”  "I never thought TB could happen in the spine. I was looking for bone pain treatment, not TB medicine." |
|  |  | Cultural beliefs | “Some even said it’s because I don’t follow proper cleanliness…”  "Eating cold rice at night causes TB, that’s what they say."  "We thought someone had done black magic. I went to a temple first". |
|  |  | Poor awareness of DR-TB | “I don’t understand—I’m scared… how can it be resistant if I never took any treatment.”  "I thought I had recovered, so I stopped the medicines."  "The doctor said my TB is resistant, but I don’t know why. I took all medicines on time before." |
|  | **1.2 Uncertain and varied interpretations of infection source** | Household exposure | “My husband had TB… I keep wondering—did I get it from him?”  “Maybe I got it while caring for my brother who had TB.”  My husband had TB for a year… I didn’t know I could get it."  "He used to cough all night. We slept in the same room." |
|  |  | Community exposure | “There are many people with TB in our village… I don’t know where I got it.” |
|  |  | Health system exposure | “We sat for hours in the hospital… I keep thinking—did I catch it that day?” |
| **Theme 2: Fragmented diagnostic pathways and uncertainty during treatment** | **2.1 Delayed and fragmented diagnostic pathways** | Missed diagnosis | “They said it's a gland disease… only later someone sent me for a TB test.”  “First they treated me for typhoid, then later said it was TB of spine.” |
|  |  | Health system delays | “I had to go three times just to get the report… by then my condition worsened.”  “Every hospital just gave me another slip for somewhere else.”  "I took first-line drugs for months before anyone checked if it was MDR."  "They didn’t do the Machine (GeneXpert) test at first. I wasted two months on wrong medicines."  "In my village, no one could diagnose my illness. I had to travel four times to the city before someone said it was TB." |
|  |  | Informal care | “I used to take antibiotics from the medical store… the pain reduced.”  “Too many tablets… I felt worse after starting medicines.”  "The local doctor gave me pills for everything. When I got worse, I came to city." |
|  | **2.2 Treatment challenges, poor communication, and uncertainty in monitoring** | Adverse drug effects | “The medicine made me vomit… I just decided to stop on my own.”  “I left the medicines and started taking Ayurvedic. I thought allopathy is worsening my disease.”  "The tablets made me dizzy and nauseous. I stopped for 10 days."  "I was vomiting every day. My ears started ringing. I felt like stopping the medicine." |
|  |  | Monitoring uncertainty | “I didn’t know if the medicines were working… there was nothing to show.” |
|  |  | Poor communication | “No one told me what drug resistant means… they just gave medicines.”  "No one explained what is my disease or what to expect."  "We only got medicines. No one explained what to expect or how to handle the side effects." |
|  |  | System gaps | “They just changed the medicine… I didn’t understand why.”  ‘Sometimes the medicines were out of stock. I had to wait for a week." |
| **Theme 3: Socioeconomic and psychosocial impact across the illness trajectory** | **3.1 Financial strain and work disruption** | Economic burden | “I couldn’t go to work… all my money was gone.”  “I had to spend from my pocket for private tests. Government hospital was far.”  "We sold our cow to continue my treatment."  "Government gives medicines, but I lost my job. I was the only earner."  "We spent 300 rupees on every visit. Sometimes I had to skip doses because I couldn’t afford the trip." |
|  |  | Dependency | “I had to borrow from neighbours… this illness breaks the whole family.”  "My mother left her job to take care of me."  "My brother says I’m wasting money and should stop the drugs." |
|  | **3.2 Emotional distress, stigma, and gendered experiences** | Stigma | “People’s behaviour changes… TB patients are seen as lesser.”  “My neighbors stopped talking after they heard I had TB.”  "They think I’m dirty. No one comes near me now."  "I told my employer it’s just bone infection. If I say TB, they will stop me from work."  "My wife helped with medicines but said not to tell neighbors." |
|  |  | Emotional distress | “I began to feel like I was different—as if I was tainted.”  “I cried every night… I felt it’s all over for me.” |
|  |  | Gendered impact | “I was worried my in-laws might not take it well… I lived in constant fear.”  "My husband left me after I told him. He said he can’t risk the children."  "My husband didn’t allow me to go to the city hospital alone. For every test, I had to wait." |
|  |  | Role-related stress | “I felt ashamed I couldn’t support my family.” |
